# Brain Gains: a literature review of medical missions to low and middle-income countries

**DOI:** 10.1186/1472-6963-12-134

**Published:** 2012-05-29

**Authors:** Alexandra LC Martiniuk, Mitra Manouchehrian, Joel A Negin, Anthony B Zwi

**Affiliations:** 1 The George Institute for Global Health Senior Research Fellow, Senior Lecturer, Faculty of Medicine, University of Sydney, Sydney, Australia; 2 Scientist, Health Systems Research, Sunnybrook Health Sciences Centre Associate Professor, Dalla Lana School of Public Health, University of Toronto, Toronto, Canada; 3 Plan International Canada, 95 St. Clair Avenue West, Suite 1001, Toronto, ON, M4V 3B5, Canada; 4 Senior Lecturer in International Public Health Lecturer in International Public Health, Sydney School of Public Health Research Fellow, Menzies Centre for Health Policy, University of Sydney, Sydney, Australia; 5 Professor of Global Health and Development, School of Social Sciences, The University of New South Wales, Sydney, NSW, Australia

**Keywords:** medical missions, low- and middle-income countries, volunteer, human resources

## Abstract

**Background:**

Healthcare professionals’ participation in short-term medical missions to low and middle income countries (LMIC) to provide healthcare has become common over the past 50 years yet little is known about the quantity and quality of these missions. The aim of this study was to review medical mission publications over 25 years to better understand missions and their potential impact on health systems in LMICs.

**Methods:**

A literature review was conducted by searching Medline for articles published from 1985–2009 about medical missions to LMICs, revealing 2512 publications. Exclusion criteria such as receiving country and mission length were applied, leaving 230 relevant articles. A data extraction sheet was used to collect information, including sending/receiving countries and funding source.

**Results:**

The majority of articles were descriptive and lacked contextual or theoretical analysis. Most missions were short-term (1 day – 1 month). The most common sending countries were the U.S. and Canada. The top destination country was Honduras, while regionally Africa received the highest number of missions. Health care professionals typically responded to presenting health needs, ranging from primary care to surgical relief. Cleft lip/palate surgeries were the next most common type of care provided.

**Conclusions:**

Based on the articles reviewed, there is significant scope for improvement in mission planning, monitoring and evaluation as well as global and/or national policies regarding foreign medical missions. To promote optimum performance by mission staff, training in such areas as cross-cultural communication and contextual realities of mission sites should be provided. With the large number of missions conducted worldwide, efforts to ensure efficacy, harmonisation with existing government programming and transparency are needed.

## Background

Globalization has led to both brain drains and brain gains of health human resources from and to low-and middle-income countries (LMICs). The number of health professionals from high income countries (HIC) on medical missions to provide care in LMICs is growing globally. Medical schools are also noting increased demand for educational electives in LMICs. For instance, in the United Kingdom a 2002 survey showed 40% of medical school students participated in a 6–8 week elective mission to a LMIC [1]. The University of California, San Francisco reports 41% of residents in their orthopaedic department alone have been involved in a medical mission to a LMIC as part of their training [2]. With the growing prevalence of these medical missions is a parallel need to understand, quantify and potentially guide, these human resource flows. A recent article in the Lancet (March 2011) highlights the increasing need for accountability and standards when health aid is offered to another country, but few data exist [3].

Thus, this paper reviews the literature on medical missions to LMIC and aims to identify the type of healthcare provided and its potential impact on the local health system and to analyse trends regarding short-term medical missions published over the past 25 years. In reviewing the literature for this study, it was revealed there is no one term strictly used for these types of visits. The literature uses a variety of terms, including ‘medical brigades’, ‘volunteer trips’, and ‘humanitarian assistance’. Publications reviewed discussed a variety of mission types, from informal one-time trips conducted by a single nurse or doctor, to highly organized repeat missions consisting of a variety of healthcare personnel, logisticians, medical equipment, and medications travelling to a region where research and evidence demonstrated a distinct need for outside medical intervention. In the context of this paper, a medical mission refers to a short trip of 1 day to 2 years by a healthcare professional to a LMIC to provide direct medical care to the population at large, or to a particular subset of the population identified by their particular health need, age group, or cultural group. Healthcare professionals participating in medical missions are typically citizens of high income countries (HIC). The diverse nature of these trips is doubtless one of the reasons for the lack of analysis, policy-making and academic discussion about medical missions. As the first literature review of short-term medical missions, this review aimed to highlight potential advantages and disadvantages of medical missions and to make recommendations regarding improved policy making, mission planning, reporting, monitoring, evaluation and future research in this area.

## Methods

This paper follows the ‘Preferred Reporting Items for Systematic reviews and Meta-Analyses’ (PRISMA) guidelines [4-6]. An Additional file 1 is provided with detailed Methods including Flow Chart of article inclusion/exclusion. Articles were identified using Medline. Articles were limited to those in English, published between August 1985 and December 2009 (25 years). Articles were separated into those which had abstracts (Group A) and those without (Group B). The titles of both groups of articles were reviewed to determine if they met inclusion criteria. Articles were eligible if they discussed medical missions to low or middle-income countries (as defined by the World Bank), and where the medical mission duration was two years or less. Where inclusion/exclusion could not be determined on the basis of article title alone, abstracts were reviewed (Group A) and full articles were reviewed for those without abstracts (Group B). In both Group A and Group B, articles were divided into three categories: ‘Relevant’, ‘Not Relevant’, and ‘Maybe Relevant’. Authors decided an article’s relevance (inclusion) by determining if the mission served LMIC residents, if the mission had direct patient contact, and by reviewing the duration and nature of medical missions. Once relevant articles were identified, and data entered into a standard extraction form (below), descriptive analyses of the data collected were conducted in Excel.

Information extracted from articles (further detail provided in supplemental methods file)

Article Information:

· Author(s)

· Year published

· Journal

· Article description – broken down into three categories: Descriptive; Critical Appraisal; Theoretical or Conceptual

· Research methods: Identification of research methodology described in article, if any

Mission Details:

Mission Type

o Exchange: Exchange of healthcare professionals between two countries

o Short-term mission: Missions which last 1 day – 4 weeks

o Medium-term mission: Missions which last 5 weeks – 6 months

. Long-term missions: Missions which last 7 months – 2 years

· Sending country

· Destination country

· Sending organization

· Receiving organization

· Mission Funding Source

· History of collaboration: Year collaboration began

Medical Aspects of Mission:

· Health professional type (i.e. surgeon, dentist, etc.)

· Type of care provided

· Disease/health issue

Education or Training

· Students involved? (Yes/No)

· Training involved? (Yes/No)

· Training details (i.e. type of training provided during mission)

Medical missions to poor communities within high income countries – for example articles which discussed post-Hurricane Katrina assistance in the U.S. or missions to Aboriginal communities in Australia – were not included [7,8]. Military medical missions were excluded. Medical missions to areas struck by a natural disaster or complex humanitarian emergency were also excluded. These were excluded as it was felt that missions to provide emergency care are driven by acute need, where a critical care response is clearly needed, and these issues were felt to differ from those of short-term (1 day to 2 year) medical missions which were the focus of this literature review. Finally, articles on missions that did not provide direct medical care to communities, for example accounts of volunteers collecting and delivering medical supplies, were excluded [9].

## Results

### Study selection

A total of 230 articles were identified for inclusion in this review. The Medline search for articles written between 1985 and 2009 revealed 2512 articles, without any duplicate articles. During the process of reviewing the articles, one additional article was found to be relevant through reading the reference lists of each article, resulting in a total of 2513 articles reviewed. Taking exclusion criteria into account, 1688 articles (67%) were discarded during the title and abstract (fully papers for those without abstracts) review process. A further 411 articles (or 16.3%) were discarded as the full text of the articles could not be found through the University of Sydney Library. Thus, the full text of 414 (16.5%), articles were reviewed in detail by author (MM) and data were recorded in the Excel data extraction form. Of these 414 articles, 163 were discarded as further review by three co-authors (AM, JN, MM) revealed they did not meet inclusion criteria. At this stage, 251 articles were left, 44 of which were subjected to further review by authors (AM, JN, MM). Of the 44 articles further reviewed by authors, 21 were discarded as they did not meet the scope of the review, providing a total of 230 articles included, or 9% of the total articles found in the original search.

### Study characteristics

**Figure 1:**
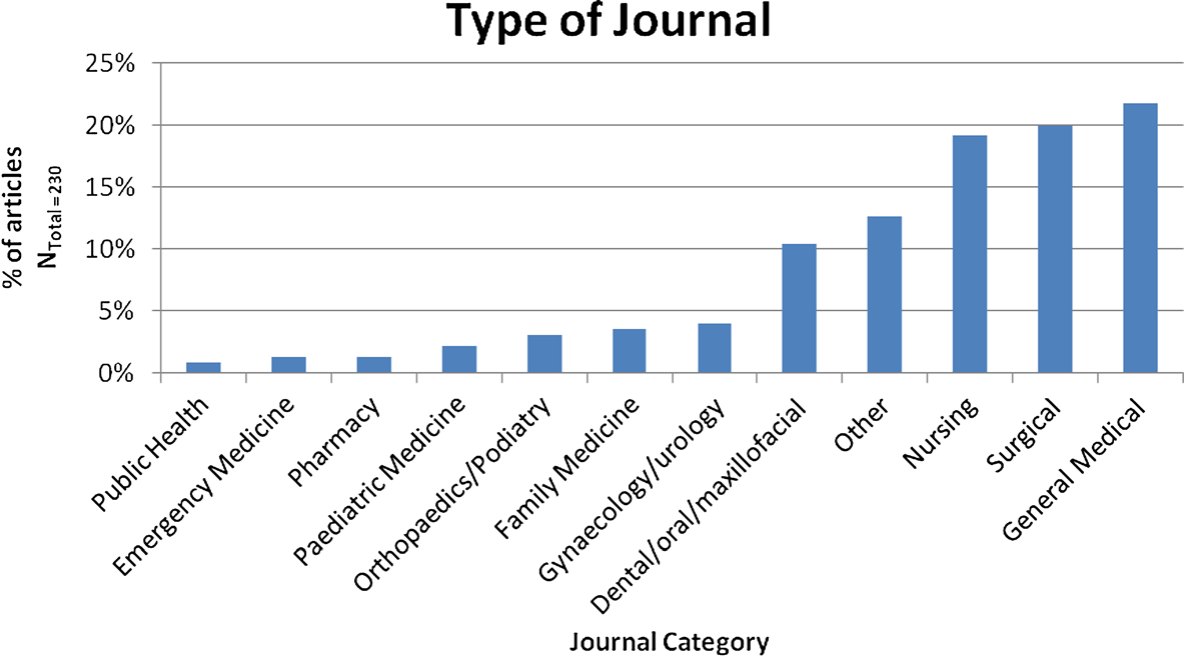
**Journal categories.** *’Other’ represents journals that did not fit into the 11 categories presented here.

Nursing, surgical and general medical journals such as the Canadian Medical Association Journal and the Medical Journal of Australia have published the highest numbers of medical mission articles (Figure 1). Articles were divided into 3 different categories: (1) descriptive articles about a medical mission but with no contextual analysis or evaluation, (2) critical appraisal articles describe a medical mission and either evaluate the mission or provide an analysis of the effectiveness of the medical mission and (3) theoretical or conceptual articles did not discuss a specific mission, but evaluate the concept of medical missions as a whole. The majority (78%) of articles identified for this study were descriptive, with only 5% having any theoretical or conceptual analysis.

The duration of medical missions was also examined. Very short-term missions were the most common, with 74% articles about medical missions which provided health care from 1 day to 4 weeks. Thirteen percent of missions were 5 weeks to 6 months in duration and the remaining proportion of articles described medical missions that were 6 months to 2 years long.

**Figure 2:**
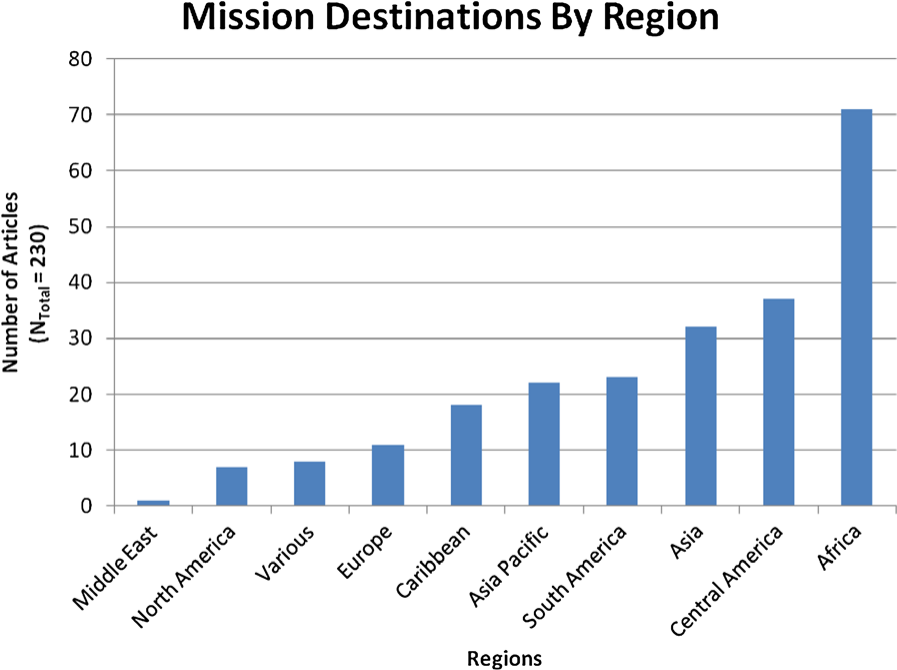
**Destination of medical missions demonstrated by region**. Note: ‘Various’ represents missions with multiple destinations.

Most health professionals engaging in short-term medical missions were from the USA. Excluding articles that did not specify sending country, the USA, Canada, Australia and the United Kingdom represent the top four sending countries for which medical mission articles have been published in the past 25 years. With regard to destination countries, the top two destination countries mentioned in publications about missions were Honduras at 6.8% and Papua New Guinea at 3.6% of all missions Figure 2. Several countries received between 1-3% of missions, including Afghanistan, Bolivia, China, Ethiopia, Haiti, India, Liberia, Mexico, Peru, Russia, Somalia, Sudan, and Uganda. Patterns existed in sending and receiving countries (Table 1). The USA sends short-term medical missions to Honduras most often, Canada to Somalia, Australia to Papua New Guinea and the United Kingdom equally to Sri Lanka, Peru, Ghana, Tanzania, and Uganda.

**Table 1:** Top four sending countries and their respective most common destination countries

**Sending Country**	**Destination Country**	**Total**	**%**
USA	Honduras	16	12.7
	Costa Rica	13	10.3
	Mexico	7	5.6
Canada	Ethiopia	3	11.5
	Somalia	3	11.5
	Chad	2	7.7
	Ecuador	2	7.7
	Guatemala	2	7.7
	Sudan	2	7.7
	Afganistan	1	3.9
	China	1	3.9
	DR Congo	1	3.9
	Ghana	1	3.9
	Haiti	1	3.9
	Israel	1	3.9
	Lesotho	1	3.9
	Malawi	1	3.9
	Nigeria	1	3.9
	Papua New Guinea	1	3.9
	Peru	1	3.9
	Tajikistan	1	3.9
	Uzbekistan	1	3.9
	Zimbabwe	1	3.9
United Kingdom	Uganda	2	11.1
	Tanzania	2	11.1
	Ghana	2	11.1
	Peru	2	11.1
	Sri Lanka	2	11.1
	Did not specify	2	11.1
	China	1	5.6
	Ethiopia	1	5.6
	India	1	5.6
	Nepal	1	5.6
	South Africa	1	5.6
	Zambia	1	5.6
Australia	Papua New Guinea	5	27.8
	Solomon Islands	3	16.7
	Afghanistan	1	5.6
	Burundi	1	5.6
	Cambodia	1	5.6
	China	1	5.6
	East Timor	1	5.6
	Kiribati	1	5.6
	Philippines	1	5.6
	Rwanda	1	5.6
	Russia	1	5.6
	Sierra Leone	1	5.6

**Figure 3:**
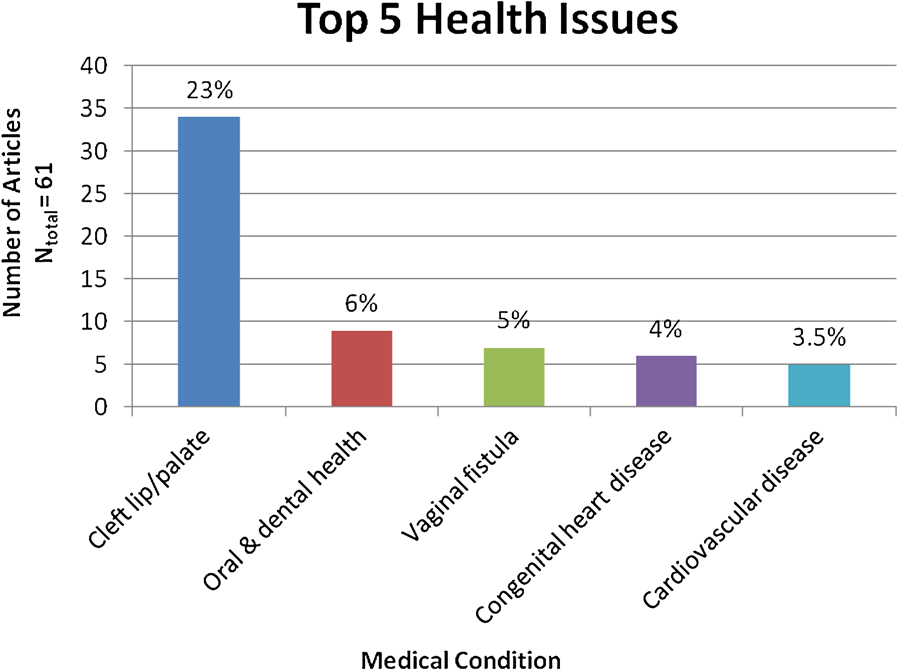
**Top five medical conditions managed on medical missions to LMICs**. *Note: excluding the “Responsive” and “Not specific” categories.

The majority of articles described short-term medical missions which assisted with the needs of patients as they arrived at the clinic/hospital. They provided ‘responsive’ care, or responded to a wide range of medical conditions, from primary care, response to injuries and severe trauma, maternal and child care, vaccine distribution, and infectious disease management. Of those missions that specified the health condition being focused upon, the most common were: cleft lip and palate deformities (23%), oral and dental health (6%), and vaginal fistulas (5%) (Figure 3).

## Discussion

### Summary of evidence

To the best of our knowledge this review of short-term medical missions is the first to assess this subject, determine trends that have developed over the past 25 years, and provide recommendations for further research and expansion of knowledge in this field. Overall, this review revealed that relatively few articles are published on the topic of short-term medical missions, in some cases, fewer than 10 per year, and publications about mission sending are dominated by four countries (USA, Canada, United Kingdom, and Australia). Nearly all articles lacked information on potential biases, such as funding source and sending and/or receiving organizations. Existing articles are mainly descriptive in nature. Very few discussed the ethics, policies, standards or evaluations of short-term medical missions. Sending countries often have a political or economic tie to the destination countries. Key medical conditions addressed by medical missions are cleft lip and palate surgeries, oral and dental health, and vaginal fistulas.

#### Benefits of medical missions

Health care professionals stated they gained a great deal from the missions and referred to missions as opportunities to reconnect to the reasons why they decided to become doctors [10]. Local community members often stated they felt medical missions demonstrated the outside world recognized their plight, and cited feeling a sense of solidarity when foreigners came to their communities to provide medical assistance: ‘they all replied that having a physician come, even for short periods of time, was extremely helpful to the community, as it put a human face on their problems and gave them hope that ongoing assistance would follow’ [11,12]. Many health care professionals on missions felt an integral part of their role was to engage in or facilitate a transfer of skills and knowledge to local counterparts [13]. This was reported through Operation Smile missions in Colombia, [14] and by the international NGO Interplast [15] both for cleft clip and palate surgeries.

#### Common critiques of medical missions

Several weaknesses of short-term medical missions were also discussed in the literature. Articles stated that foreign-led medical missions, while providing some short-term relief and aid to communities in LMICs, were ultimately not sustainable [16]. Articles also conveyed a strong sense of the limited impact of medical missions Kasis et al. [17]. One participant, in writing about a mission to Honduras questions the efficacy of these types of short term missions: ‘I can't help wondering however, that even though we really helped many of the people, for others all we really did was put a band-aid on a gaping wound. Now that we are gone will the wound just grow larger and larger?’ [18]. Many others question if medical missions are an appropriate allocation of already scarce resources, both financial and human [11,13,19-21]. Maki sums up the problems of some medical missions when they state: ‘Paucity of follow-up data, poor relations with the local health care system, and lack of sustainability can challenge the good intentions of missions’ [22].

Tied in with the question of sustainability of medical missions is the question of cost-effectiveness. This is difficult to assess since most articles do not report how much missions cost or how they are funded [23]. In view of the considerable costs involved in financing medical missions (airfare, accommodations, vaccinations, visa costs, customs fees for medicines and medical equipment etc.), it is often asked if money would be better spent donated directly to healthcare facilities in the destination country [24]. When sharing accounts of his medical mission to Zimbabwe, Buchman wonders if ‘the money that was spent on my stay could have been better spent on medical equipment, medications, or even basics such as food and housing’ [11]. Abdullah asks “what business did our team of 10 members (have in doing this, given the 10 members) have spent approximately $30,000 toward travel and hotel costs…. when the entire cost of building a new 30-bed wing for the hospital in Ghana was $60,000?” [13].

Of likely concern is the quality and efficacy of the medical care provided by foreign doctors who can be unfamiliar with local health needs, local culture and the strengths and limitations of the healthcare system in which they must leave their patients for follow up care. Doctors who are not qualified for a particular type of surgery in their home countries are often placed in situations during medical missions where they must provide care for which they are neither qualified nor confident to provide [25,26]. Trainee doctors and surgeons may not receive the typical senior supervision they may have at ‘home’ while attempting procedures with which they are unfamiliar. This may result in patients developing serious medical complications and local doctors developing strong feelings of resentment towards medical missions [27]. However, it may also be argued that this may be the best potential care that exists for a patient in a particular location, at a particular time.

There are also accounts of surgeons participating in medical missions for reasons termed ‘surgical tourism’. As certain conditions are rarely seen in high income countries, doctors are choosing to volunteer for medical missions to hone skills and see conditions which they might not otherwise encounter [10,24,28,29]. One author describes with relish ‘What we read about in books during our residencies walks in the door…It is a veritable feast of interesting cases’ [30]. In his 2006 article on medical missions to LMICs for the surgical repair of vaginal fistulas, Wall states ‘such projects may serve to promote ‘fistula tourism’ rather than significant improvements in the medical infrastructure of the countries where these problems exist’ [31].

Medical missions are often unable to provide the full-spectrum of care required for complex medical conditions. Patients with cleft lip and palate conditions, for example, need oral/maxillofacial surgeons for the initial surgical repair of the cleft lip/palate, with more post-surgical care often required. Patients can require follow-up visits to general physicians and/or plastic surgeons, future visits to orthodontists to repair damaged teeth and jaws, and possibly speech therapists to improve challenges with speech – care which they are unlikely to receive in their communities after the medical mission team departs [32,33]. Zbar et al. [34] state ‘during the past three decades, it has become increasingly clear that successful cleft management requires a multidisciplinary, long-term, team approach. To send a cleft surgeon to a remote region of the world without consideration of a genetic, dental, speech, or hearing evaluation of the patient population is perhaps irresponsible or, at best, purely an aesthetic rather than functional undertaking,’ demonstrating the deficiencies of at least some short-term medical missions in fully addressing needs of patients in LMICs [34].

#### Attitudes of healthcare professionals on mission

Many health care professionals were not aware of the depth of poverty or limits of medical facilities in the regions they were visiting [35] and had little knowledge of the local social, economic, political contexts. One student on a short-term medical mission writes ‘I knew I was going to an area of extreme poverty, but as I looked at the conditions in which the family lived, I was not quite prepared for the reality of true poverty’ [36]. This lack of awareness about the realities in LMICs often manifests itself by authors using inappropriate language that is insensitive to the local context and demonstrates a lack of respect for local health care professionals. For example, in this excerpt the author tries to explain differences in working conditions between the USA and Guatemala as staff having inadequate knowledge rather than a lack of resources: “Universal precautions were an unfamiliar concept…At the end of each day, the hospital staff would go through our trash and sharps containers, pulling out items that they could sterilize and use again” [37].

#### Political ties between sending and destination countries

Countries send missions to countries with which they have pre-existing political relationships. For example, the USA sends the majority of its missions to Honduras as well as Nicaragua, two countries with which it has significant socio-political ties. This pattern has been well documented in previous research. For instance, in their review of foreign aid distribution, Alesina & Dollar analysed the flow of bilateral aid reported by the Organization for Economic Cooperation and Development (OECD) and found a strong correlation between colonial status and the amount of foreign aid received [38].

#### Medical missions often treat rather than prevent conditions

During short-term medical missions, health care professionals often treat individuals with illnesses which could be prevented if detected earlier, but as people have little access to health care, illnesses arise and also become more severe and difficult to treat [39]. Simply responding to the needs of the patient, while reducing individual suffering, does not address the health needs of the community as a whole. Prevention such as safe water, immunization, insecticide-treated bed nets for malaria, prevention of mother-to-child transmission of HIV, or seatbelts to reduce motor vehicle injury are more likely to reduce the burden of disease in a community. However, often due to scant financial and human resources locally and a lack of interest in delivering preventative programs by foreign, visiting short-term medical mission volunteers – missions are left treating illnesses rather than preventing them.

### Strengths and limitations

This is among the first literature reviews of medical missions. Short-term medical missions have no internationally agreed upon definition and are rather difficult to define. This review limited included articles to those written in English and did not include articles describing long-term medical care (ie longer than 2 years), education or capacity development, missions occurring in a defined time period related to a complex humanitarian emergency or medical care provided to local citizens by a foreign military presence. Exclusion of these articles may have limited the discussion of medical missions overall but given their differences in reasons and length of engagement compared to short-term medical missions these were excluded to help retain the focus of this review. Only MEDLINE-indexed articles were searched as the feasibility of systematically addressing all grey literature was not possible.

### Risk of bias in individual studies and across studies

The majority of articles reviewed in this study were descriptive there was little quantitative data to analyse. The articles included in this review were typically written by a health care professional participating in the mission and thus the articles are likely subject to several types of bias including selection bias and observer bias. This study was limited to articles which were available from University of Sydney Library. Publication bias may exist in the review of this literature. Articles may not have been published if they are too critical or portray missions negatively. In reviewing all 230 articles, most articles did not list all the information which this study was attempting to gather. The information which was most often absent was the funding source. Fifty six percent of the publications examined did not include how their mission to LMICs was funded.

## Conclusions

In an increasingly globalized world, it is unlikely that the phenomenon of medical missions will diminish in the near future [40]. More information on this topic is needed. This review should be used to catalyse further discussion on all aspects of medical missions, from implications of missions on a country’s health policy and human resources for health, to the ethics of spending money to support medical missions versus spending money to further develop the health systems of LMICs. The implementation policies of medical missions and the ethics of sending health care professionals from high-income countries where training is not often in concert with the skills required upon arrival in the destination country must also be investigated further. Although the engagement of health professionals from high-income countries with people in LMIC has improved over the 25 years included in this review in keeping with the recent Paris Declaration, many questions remain about short-term medical missions specifically including: have long-term missions had an impact on the healthcare system of specific countries, particularly countries which have been receiving missions for a number of years? Do countries know who departs/arrives to provide short-term medical care? Do short-term medical missions refer patients back into the local system for follow-up care? What impacts have medical missions had on students (i.e. medical/dentistry/nursing students), both foreign and in destination countries? What are the ethical obligations of medical missions to ensure follow-up care for their patients? What impact do short-term medical missions have on local pharmaceutical distribution systems or other on-going care systems?

For medical missions to succeed and to have greater impact over the longer term, a key recommendation of this paper is to encourage mission organizers and participants to adopt a more precise approach to mission planning, implementation and reporting. Along with reporting on financial aspects of the mission, organizers should report on number of people treated, follow-up needed and how this will occur, cost per beneficiary [23], training of local counterparts conducted and challenges faced. Health care professionals participating in medical missions should receive extensive pre-departure training, specifically in areas as social, political, economic realities of the area to which they will be sent, local language training, and cross-cultural communication. Furthermore, health care professionals with the appropriate skills and experience to specifically address the identified needs of a community should be recruited and sent to areas of need based on local decision-making. Initial steps are recently heading in this direction, as discussed in a March 2011 article in the Lancet [3] describing a formal register of health care professionals in the UK who are willing to provide care overseas. For longer term responses in the UK there is the International Health Links Centre’s Humanitarian Response Register and the more recent, short-term response register has been established with the UK Government’s Department of Health and Department of International Development together with non-governmental organizations including Medical Emergency Relief International (Merlin) [3].

## Competing interests

The authors declare that they have no competing interests.

## Authors’ contributions

AM conceived of the study idea, reviewed articles, reviewed drafts of the manuscript and prepared the final manuscript. MM conducted the literature search, applied inclusion and exclusion criteria, extracted data from articles and drafted initial manuscript. JN reviewed articles, and reviewed and approved the final draft of the manuscript. AZ reviewed drafts of the manuscript. All authors read and approved the final manuscript.

## Pre-publication history

The pre-publication history for this paper can be accessed here:

http://www.biomedcentral.com/1472-6963/12/134/prepub

## Supplementary Material

Additional file 1: Detailed Methods of Literature Review addressing each of the PRISMA Guidelines. Click here for file
