# Supplementary material for: Brain Gains: a literature review of medical missions to low and middle-income countries
Source: BMC Health Serv Res. 2012 May 29;12:134. doi: 10.1186/1472-6963-12-134 (PMC3474169; doi:10.1186/1472-6963-12-134)
Supplement: Additional file 1: — Detailed Methods of Literature Review addressing each of the PRISMA Guidelines. [file 1472-6963-12-134-S1.docx]

Oct 15 2011

**Brain Gains: a literature review of medical missions to low and middle-income countries**

Martiniuk ALC, Manouchehrian M, Negin J, Zwi A

**Supplemental File:**

**Detailed Methods of Literature Review addressing each of the PRISMA Guidelines**

**Protocol and Registration**

None

**Eligibility criteria**

Inclusion criteria:

Articles were eligible if they were written in English and published in a MEDLINE-indexed journal between 1985 and 2009 and discussed a medical mission to provide direct patient care in a low or middle income country. Since the focus of this review was on short medical missions, articles were eligible for inclusion where the duration of the medical mission was two years or less. Low and middle income countries were identified using the World Bank 2009 classification, “World Bank List of Economies 2009” (The World Bank 2009). Medical missions to poor communities within high income countries – for example articles which discussed post-Hurricane Katrina assistance in the USA or missions to Aboriginal communities in Australia – were not included (Bradley 1999; Holbrook 2006).

Exclusion criteria:

Articles were excluded if the medical mission was being undertaken by the military of a government. Medical missions to areas struck by a natural disaster or other humanitarian emergency were also excluded. These were excluded since it was decided that these medical missions are driven by acute need, where a critical care response is required.

Medical missions which did not involve direct patient contact were also excluded. Thus, articles which, for example, provided accounts of volunteers collecting and delivering medical supplies but who did not provide direct medical care to communities, were excluded (Rollins 2004). As well, articles focused solely on capacity building, education, institutional strengthening, or research system support were excluded from this review on medical missions providing short-term medical care.

**Information sources**

Articles were identified through MEDLINE as well as hand searching the reference lists of each of the included articles. One additional article was identified through reviewing reference lists.

**Search**

MEDLINE-indexed articles were searched according to the search criteria detailed in Box #1. Figure 2 is a Flowchart demonstrating the inclusion of articles from Step 1 to Step 7.

**Figure 1 (Box 1):** search parameters

**Box 1: Search terms**

("1985/08/01"[PDAT]: "3000"[PDAT]) AND (("brigade"[All Fields] OR (("mission"[All Fields] AND "short-term"[All Fields]) OR ("mission"[All Fields] AND "overseas"[All Fields]) OR ("mission"[All Fields] AND "foreign"[All Fields])) OR (("trip"[All Fields] AND "short-term"[All Fields]) OR ("trip"[All Fields] AND "overseas"[All Fields]) OR ("trip"[All Fields] AND "foreign"[All Fields]))) OR (("mission"[All Fields] AND "volunteer"[All Fields]) OR ("trip"[All Fields] AND "volunteer"[All Fields]) OR ("short-term"[All Fields] AND "volunteer"[All Fields]) OR ("overseas"[All Fields] AND "volunteer"[All Fields]) OR ("foreign"[All Fields] AND "volunteer"[All Fields])) OR (("foreign"[All Fields] AND "humanitarian"[All Fields]) OR ("short-term"[All Fields] AND "humanitarian"[All Fields]) OR ("overseas"[All Fields] AND "humanitarian"[All Fields])) OR ("medical mission"[All Fields] OR "medical missions"[All Fields]) OR ("medical tourism"[All Fields] OR "health tourism"[All Fields] OR ("tourism"[All Fields] AND "medical"[All Fields])) OR (("assistance"[All Fields] AND "short-term"[All Fields]) OR ("assistance"[All Fields] AND "overseas"[All Fields]) OR ("assistance"[All Fields] AND "foreign"[All Fields]) OR ("assistance"[All Fields] AND "humanitarian"[All Fields]) OR ("assistance"[All Fields] AND "volunteer"[All Fields]))) AND ("humans"[MeSH Terms] AND English[lang])

**Study selection**

Eligibility of potentially relevant articles was assessed throughout the entire review process (Figure– Flowchart). At the end of the process (Step 6 in Figure), articles that did not strictly meet inclusion criteria were reviewed by (AM, JN) and final decisions regarding inclusion were made by (MM, AM, JN).

**Data collection process**

Data was extracted by one co-author (MM) from all reviewed articles and entered into an Excel spreadsheet. Data extraction was reviewed by two co-authors (AM, JN) and additional data retrieved from the article where necessary.

Articles retrieved from the initial search were first separated into those which had abstracts (Group A) and those without (Group B). Titles were reviewed to determine if they met with inclusion criteria. Abstracts were reviewed where the content of the article was not able to be determined from the title alone, abstracts were reviewed for those with abstracts (Group A) and full articles were reviewed for those without an abstract (Group B).

During abstract review (or full article review where no abstract existed) articles were sorted (by MM) into three categories based on *apriori* inclusion criteria: “Relevant”, “Not Relevant”, and “Maybe Relevant”. The three categories were then reviewed by two other co-authors (AM, JN). The “Not Relevant” articles were not reviewed any further. Full articles were obtained from the University of Sydney library for all “Relevant” and “Maybe Relevant” articles. According to the Australian Government Department of Education, Employment and Workplace Relations, The University of Sydney has one of the largest library holdings in the world (<http://www.dest.gov.au/> Cited: 15/10/2011).

Full articles were reviewed for inclusion. During this review, data from each article were extracted and entered into the Data Extraction form using Excel. After entry of available data from each of the articles included at this stage, articles were again sorted into “Relevant”, “Not Relevant” and “Unsure” categories. Articles and their extracted data, in the “Unsure” category were reviewed by three of the co-authors (AM, JN, MM) to determine final inclusion or exclusion of the article.

**Data items**

From each study the following information was extracted (where available in the published article) and entered into an Excel spreadsheet.

Article Information:

- Author(s)
- Author institution(s)
- Year published
- Article title
- Journal
- Article type – as categorized by Medline(U.S. National Library of Medicine - National Institutes of Health 2009)
- Article description – categorized into three categories:
  - Descriptive: Articles which simply describe a medical mission and provide no other contextual analysis or evaluation
  - Critical Appraisal: Articles which describe a medical mission and either evaluate the mission or provide an analysis of the effectiveness of medical missions
  - Theoretical or Conceptual: Articles which do not discuss a specific mission, but which evaluate the concept of medical missions as a whole
- Research methods: Identification of research methodology described in article, if any
- Article subject - grouped into five categories:
  - Implementation: Description of the medical mission listing details such as who went on mission, length of mission, goals etc.
  - Ethics: Articles which discussed and/or evaluated the ethics of bringing in foreign doctors, supplies etc. to low and middle income countries
  - Policy: Articles which discussed the health policy, human resources for health policy, or implementation policy implications of medical missions
  - Theory: Articles which did not discuss a particular mission, but which provided ideas on how best to manage or implement missions
  - Not Applicable (N/A): None of the above subjects were discussed

Mission Details:

- Mission Length – Exact length of mission listed, if provided, e.g. number of days, months, years
- Mission Type – Level of collaboration, sorted into five categories:
  - Exchange: Exchange of health care professionals between two countries, one of which is a low or middle income country
  - Short-term mission: Missions which last 1 day – 4 weeks
  - Medium-term mission: Missions which last 5 weeks – 6 months
  - Long-term missions: Missions which last 7 months – 2 years
  - Not applicable (N/A): Article did not specify mission length, or article does not discuss a particular mission (i.e. can be a Theoretical or Conceptual article)
- Sending country
- Destination country
- Sending organization: If the health care professional went on mission through an intermediary organization, this information was collected and categorized as one or several of:
  - Non-governmental organization (NGO)
  - Community-based organization
  - Religious organization
  - University
  - Hospital
  - Medical Association
  - Governmental organization
  - Self-organized
  - Not applicable (N/A) – either no mention of how the health care professional arranged overseas trip or article does not discuss specific mission
- Receiving organizations: where applicable, recorded what type of organization welcomed and hosted the volunteers. Categories are broken down as with Sending Organization
- Mission Funding Source:
  - Corporate funding
  - Non-governmental organization (NGO)
  - Community-based organization
  - Religious organization
  - University
  - Hospital
  - Medical Association
  - Government-funded
  - Self-funded
  - Fundraising
  - Not applicable (N/A) – either no mention of funding source or article does not discuss specific mission
- History of collaboration: Year collaboration began

Medical Aspects of Mission:

- Health professional type (i.e. surgeon, dentist etc.)
- Type of care provided
- Disease/health issue

Education or Training

- Students involved? (Yes/No)
- Student details (i.e. type of student)
- Training involved? (Yes/No)
- Training details (i.e. type of training provided during mission)

**Bias in studies**

Studies were qualitative in nature and thus bias is inherent.

**Summary measures**

As studies were qualitative in nature, no summary measures were calculated.

**Synthesis of results**

Quantitative descriptive analyses were conducted for included studies using Excel.

Medical Missions in Low and Middle Income Countries: A Literature Review
